# *Matricaria chamomilla* L. Ameliorates Asthma by Protecting OVA‐Induced Rats and LPS‐Induced Human Bronchial Epithelial Cells Through Suppressing Autophagy and Apoptosis

**DOI:** 10.1002/fsn3.70030

**Published:** 2025-02-13

**Authors:** Jun Peng, Feicui Zhao, Xiaolong Kang, Nadire Aierken, Qian Li

**Affiliations:** ^1^ Department of Medicine Research Hospital of Traditional Chinese Medicine Affiliated to Xinjiang Medical University Urumqi People's Republic of China; ^2^ Xinjiang Key Laboratory of Processing and Research of Traditional Chinese Medicine Urumqi People's Republic of China; ^3^ The Fourth Clinical Medical College of Xinjiang Medical University Urumqi People's Republic of China

**Keywords:** apoptosis, asthma, autophagy, *Matricarla chamomilla* L.

## Abstract

The rising prevalence of asthma has heightened awareness of the benefits of functional foods and nutraceuticals for managing the condition. *Matricaria chamomilla* L., a plant with various health benefits, is commonly consumed as tea in China and other countries. We previously reported the chemical composition and anti‐asthma effect of the active fraction of *M. Chamomile* (MC). This study investigated the protective mechanism of MC on asthma using an ovalbumin (OVA)‐induced asthma model in rats and lipopolysaccharide (LPS)‐induced human bronchial epithelial cells (16HBE). The effect of MC on asthmatic rats was evaluated through biochemical and histological analyses. Following treatment with MC in OVA‐induced asthmatic rats, improvements were observed in behavioral measures, total and differential cell counts of leukocytes in bronchoalveolar lavage fluid (BALF), inflammatory cell infiltration, and the structural integrity of lung and bronchial tissues. Additionally, immunohistochemical analysis revealed an increase in the protein expression level of Kif3a, while the expression levels of LC‐3B, BECN1, and Caspase‐3 were decreased. Furthermore, the effect of MC on autophagy was analyzed using an LPS‐induced 16HBE cell model. MC reduced cell damage and determined the optimal treatment concentration at 200 μg/mL for 48 h; LPS‐induced cell apoptosis was reversed by MC using flow cytometry analysis. Autophagy flux was measured through mRFP‐GFP‐LC3 adenovirus, and MC blocked the autophagic flux of 16HBE cells induced by LPS. The mRNA and protein expression of LC3‐II, BECN1, and Cleaved Caspase‐3 were decreased, whereas Kif3a was increased following treatment with MC. The protective effect of co‐treatment with 3‐MA and MC was more significant, and MC exhibited similar efficacy to 3‐MA in inhibiting autophagy. Hence, MC is a potential autophagy inhibitor, which could inhibit over‐activated autophagy levels to enhance Kif3a expression, thereby decreasing apoptosis to against asthma. *M. Chamomile* is a promising pharmaceutical and dietary supplement candidate for the amelioration of asthma.

## Graphical abstract

*Matricarla chamomilla* L. is a potential candidate pharmaceutical and food supplement for improving asthma, which exerts an anti‐asthmatic effect via inhibiting over‐activated autophagy and apoptosis in the OVA‐induced asthmatic rats model and LPS‐induced 16HBE cells model.

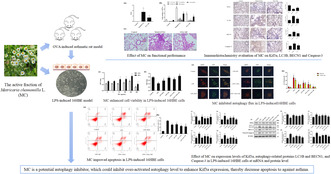

## 1 Introduction

Asthma is a common chronic inflammatory respiratory disease, which is characterized by recurrent wheezing, shortness of breath, chest tightness, and coughing. It has been a major public health challenge in the world, contributing to serious economic and social burden. More than 330 million children and adults worldwide suffer from this disease, and the number of people affected will increase to 400 million by 2025 (Chakaya and Aït‐Khaled 2022). Glucocorticoids and bronchodilators are the drugs most commonly used in the treatment of asthma, but long‐term use may give rise to adverse effects (Li and Liu 2020). Recently, using natural bioactivities from natural plant resources with anti‐inflammatory effects and low toxicity as nutritional supplements and/or pharmaceuticals would be a good alternative to improving asthma (Carrozza et al. 2023; Chou et al. 2023).

*Matricarla chamomilla* L., belonging to Asteraceae (Compositae) family, is native to Europe and Western Asia and widely cultivated in China, especially in Xinjiang (El Mihyaoui et al. 2022; Janarny, Gunathilake, and Ranaweera 2021). It is not only ornamental but also has edible and medicinal value. *M. chamomilla* is used in scented tea and chinese medicinal herbs, which have the biological activities of antioxidant, anti‐inflammatory, anti‐bacterial, anti‐depressant, anti‐cancer, anti‐diabetes, etc. (Al‐Dabbagh et al. 2019; Cvetanovic et al. 2015; Neves et al. 2009; Singh et al. 2011). Its utilization has a long history in Iranian traditional medicine in the form of tea. Besides, *M. chamomilla* is one of the main medicinal materials of Xinjiang's traditional famous prescription Zukamu granules for treating respiratory diseases (Yin et al. 2019). Our group has found that n‐butanol extract of *M. chamomile* expressed good anti‐inflammation and anti‐asthma effects (Li, Lu, and Li 2017). The anti‐asthma active component of *M. chamomile* (MC) was obtained by optimizing the process (Fei et al. 2024). The main chemical components were identified using the UHPLC‐Q‐Orbitrap‐HRMS coupling technique, which showed that polyphenol compounds were effective components against asthma, especially flavonoids and caffeoylquinic acid, were the important bioactive components, mainly including luteolin, quercetin, kaempferol, chlorogenic acid, and their related compounds (Li et al. 2023). Furthermore, our group found that MC can reduce the number of EOS in Penh and bronchoalveolar lavage fluid (BALF) and the IgE level and increase glutathione peroxidase (GSH‐Px) in the serum of OVA‐induced rats and ameliorate the asthma symptoms of OVA‐induced rats (Li et al. 2023). Therefore, MC exhibits anti‐asthmatic properties. Nonetheless, the underlying mechanisms through which it ameliorates asthma symptoms have yet to be elucidated.

The pathogenesis of asthma is complicated and includes genetic factors, immune regulation, endocrine regulation, and environmental factors. Our previous study revealed that *M. chamomile* exerts an anti‐asthmatic effect by targeting the protein Kif3a (Chang et al. 2020). Kinesin family member 3A (Kif3a), a subunit of heterotrimeric kinesin 2, regulates microtubule function and transport, which plays an important role in the pathogenesis of asthma (Xu et al. 2016). Kif3a is a key factor in the lung function of formation and maintenance of cilia in the clearance of mucociliary cells, which can affect the morphology, homeostasis, and repair function of lung cells through multiple signaling pathways involving autophagy and apoptosis (Geng et al. 2018; Xu et al. 2016). This target is a possible mechanism in the regulation of airway inflammation and a new target for the treatment of asthma. Autophagy and apoptosis are important cellular processes that maintain cellular homeostasis (Huang et al. 2022). Autophagy is an evolutionarily conserved catabolic process, and autophagy‐related genes (Atgs) play essential roles in airway epithelial regeneration and lung development (Dong et al. 2023; Jiao et al. 2021). There is growing evidence that abnormalities in autophagy contribute to the pathogenesis of asthma (Barnes, Baker, and Donnelly 2022; Lv, Li, and Hu 2020). The experiment in OVA‐induced mouse models of asthma and airway epithelial cells showed that targeted inhibition of autophagy can improve airway mucosal accumulation and airway inflammation to reduce lung tissue damage (Huang et al. 2022; Xu et al. 2018). Besides, the imbalance of apoptosis may play an important role in the process of airway inflammation of asthma (Long et al. 2023).

In this study, we established the OVA‐induced asthma rat model and LPS‐induced human bronchial epithelial cells (16HBE) to explore the effect and mechanism of MC in treating asthma. This study provides a basis for further research and application of *M. chamomile* to be used as a potential functional food and candidate pharmaceutical to treat asthma.

## 2 Materials and Methods

### 2.1 Drugs and Reagents

The human bronchial epithelial cell lines (16HBE) and the specific culture medium (CM‐0249) were purchased from Procell (China). The antibiotic (penicillin and streptomycin) was obtained from Gibco (USA). Fetal bovine serum (FBS) was obtained from Excell Bio (China). Lipopolysaccharide from *E. coli* O111: B4 (L2630) and Dexamethasone were purchased from Sigma (USA). The RFP‐GFP‐LC3 double fluorescence lentivirus was obtained from Genechem (China). 3‐Methyladenine was purchased from MCE (China). The Annexin V‐FITC Apoptosis Staining/Detection Kit was obtained from Abcam (UK). TRIzol reagent (15596026) was purchased from Ambion, cDNA reverse transcription kit (5X All‐In‐One RT MasterMix) and RIPA Lysis Buffer (AR0105) obtained from Boster (China). Easy II Protein Quantitative Kit (BCA) from TransGen Biotech (China).

KIF3A (D7G3) Rabbit antibody (#8507) was obtained from Cell Signaling Technology (USA). Anti‐LC3B antibody (ab192890), anti‐Beclin 1 antibody (ab207612), and anti‐Caspase‐3 antibody (ab184787) were purchased from Abcam (UK).

### 2.2 Plant Material

*M. Chamomila* was harvested from Yili Kazakh Autonomous Prefecture in the Xinjiang Uygur Autonomous Region, China. It was identified by Dr. Yonghe Li from the Xinjiang Uygur Autonomous Region Hospital of Traditional Chinese Medicine. Fresh *M. Chamomile* was air‐dried at room temperature.

### 2.3 Preparation of the Extracts

The active fraction of *M. Chamomile* (MC) was prepared by the reflux extraction method and macroporous resin (AB‐8 type macroporous adsorption resin) (Li, Xin, and Haji Akber 2021). For extraction condition, the ethanol concentration, time, and solid–liquid ratio were 70%, 1 h, and 1:10 (g/mL), respectively. For purification conditions, sample solution mass concentration was 0.20 g/mL (equivalent to natural drugs), the velocity of absorption was 2 BV/h, absorption was 7 BV, and then MC was eluted by 3 BV 50% ethanol and 1 BV 70% ethanol (Li et al. 2023).

### 2.4 Animal Experiments

Male Sprague Dawley rats, aged 6–8 weeks, were obtained from Beijing SBF Biotechnology Co. Ltd. They were kept in SPF facilities with unrestricted access to standard feed and water in a controlled environment of 20°C–25°C and a 12‐h light/dark cycle. All experiments followed the guidelines of the Institutional Animal Care and Use Committee of Xinjiang Medical University. The study involved 24 rats divided into three groups of eight: control, model (OVA), and OVA+MC groups.

In the experimental protocol, the sensitized animals, designated as the model group, received intraperitoneal injections of 1 mL of an ovalbumin (OVA) suspension, comprising 1 mg of OVA and 200 mg of aluminum hydroxide gel, on days 1 and 8. In contrast, the normal control group was administered an equivalent volume of saline as a control measure. One week subsequent to the second sensitization, the rats were exposed to an ultrasonic nebulizer beginning on the 15th day. They were subjected to daily stimulation with an aerosolized saline solution containing 1% OVA, delivered via the ultrasonic nebulizer for a duration of 28 days, with each session lasting 30 min. The rats in the normal control group were nebulized with an equivalent volume of saline. Following stimulation, the rats were monitored for indicators of asthma attacks, including irritability, sneezing, coughing, nasal scratching, incontinence, increased respiratory amplitude, wheezing, and cyanosis. The asthma model was deemed successful (Li et al. 2023). From day 43 to day 72, the control and model groups were administered saline via gavage, whereas the MC group received an oral dose of MC at 0.18 g/kg (Li et al. 2023).

### 2.5 Behavioral Observation

The evaluation of the OVA‐induced allergic asthma rat model's behavior was conducted 4 weeks post‐treatment, utilizing the “Asthma Attack Scale” alongside previous references (Li et al. 2023). The assessment criteria included the presence or absence of symptoms such as cyanosis, wheezing, shortness of breath, slow movement or twisting, and agitation. The scoring system was defined as follows: 0 points for normal behavior or mild shortness of breath; 1 point for trembling or nodding; 2 points for coughing, pronounced shortness of breath, restlessness, and cyanosis; 3 points for rhythmic retracted wheezing; and 6 points for severe respiratory distress.

### 2.6 Collection Total and Differential Cell Counts of Leukocytes in Bronchoalveolar Lavage Fluid (BALF)

After blood was taken from the abdominal aorta of the rat, BALF was collected by cannulation of the trachea (Li et al. 2023). briefly, the skin of the neck was cut to expose the trachea, the chest was opened to expose both lungs, and the right main bronchus was first ligation. Then 4 mL of sterile saline was dripped into the left lung, and the supernatant was recovered and centrifuged at 700 ***g*** for 10 min at 4°C, and cells were submitted to Wright‐Giemsa staining. The total number of cells, eosinophilia, lymphocytes, macrophages, and neutrophils was obtained by microscopic counting.

### 2.7 Histopathological Evaluation

Following the BALF collection, the right lung specimens from the rats were fixed in 10% neutral buffered formalin, embedded in paraffin wax, and sectioned into 3 μm slices (Leica, RM2235). The sections were subsequently stained with hematoxylin and eosin (H&E) and examined under a light microscope at high magnification (100×) (Leica BX43, Germany) to assess histopathology and inflammatory cell infiltration.

### 2.8 Immunohistochemistry Evaluation

Immunohistochemical analysis was conducted to assess the expression levels of Kif3a, LC3B, BECN1, and Caspase‐3 in lung tissue samples. Initially, lung tissue sections intended for histopathological examination were fixed and then underwent antigen retrieval through heating in a sodium citrate solution until boiling. Subsequently, the sections were incubated with 5% bovine serum albumin (BSA) for 30 min at 37°C, followed by a rinsing step. To eliminate endogenous peroxidase activity, sections are treated with 3% hydrogen peroxide in deionized water for 10 min, then rinsed with phosphate‐buffered saline (PBS). The primary antibody was incubated overnight, and then the secondary antibody was incubated for 1 h. After applying the Streptavidin‐Biotin Complex (SABC) for 20 min at 37°C and staining with DAB for 3 min, sections are stained with hematoxylin for 3 min, washed, sealed with neutral resin, and observed under a light microscope at 100× magnification to assess protein changes.

### 2.9 Cell Culture and Establishment of Inflammatory Injury Model

The human bronchial epithelial cells (16HBE) were cultured in Keratinocyte Medium (KM). The 10% fetal bovine serum (FBS) and 1% penicillin/streptomycin were added in medium. 16HBE cells were treated with LPS (2.5 μM) for 16 h at standard cell culture conditions (37°C, 5% CO_2_) to establish the LPS‐induced 16HBE cell model.

### 2.10 CCK‐8 Assay

Cell counting kit‐8 (CCK‐8) assay (TransGen Biotech, China) was performed to evaluate 16HBE cell viability. In brief, cells were seeded into a 96‐well plate (100 μL/well) at a concentration of the single‐cell suspension of 5 × 10^4^ cells/mL and cultured overnight. After induced by LPS (2.5 μg/mL, 16 h) to establish an inflammatory injury model, various concentrations (0, 1, 10, 100, 200, and 400 μg/mL) of MC were treated for 24 and 48 h, respectively. At the end of treatment, the supernatant was removed, and 100 μL of 10% CCK‐8 solution (5 mg/mL, DMSO dissolved; Beyotime, Beijing, China) was added for 1 h. Absorbance (450 nm) was measured using a microplate reader.

### 2.11 Experimental Grouping and Intervention

3‐MA was an autophagy inhibitor, and dexamethasone (DEX) was a positive drug. The cells were divided into six groups, including the control group, LPS group (model group), LPS + DEX group, LPS + MC group, LPS + 3‐MA group, and LPS + 3‐MA + MC group. The control group was cultured under normal conditions. 16HBE cells treated with 2.5 μM LPS for 16 h were considered the LPS group (model group) at 37°C in a humidified atmosphere containing 5% CO_2_. LPS‐induced 16HBE cells were subsequently treated with 1 μM dexamethasone for 2 h, which was the same condition as the LPS + DEX group. Following the induction of 16HBE cells with LPS (2.5 μM) for 16 h, the cells were subsequently treated with MC (200 μg/mL) for 48 h, constituting the LPS + MC group. The LPS + 3‐MA group involved treating 16HBE cells with LPS (2.5 μM) for 16 h, followed by 3‐MA (5 mM) for 4 h. In the LPS + 3‐MA + MC group, 16HBE cells were treated with 2.5 μM LPS for 16 h, followed by 5 mM 3‐MA for 4 h, and then 200 μg/mL MC for 48 h.

### 2.12 Flow Cytometry Assay

The apoptosis rate of 16HBE cells was detected using flow cytometry equipment (BD, USA) and the Annexin V‐FITC Apoptosis Staining/Detection Kit, according to the reference (Mota et al. 2020). In short, after being induced by LPS, 16HBE cells were treated with MC (200 μg/mL) for 48 h; these cells were resuspended in 500 μL 1× Binding Buffer after PBS washing and stained with V‐PE (5 μL) and 7‐AAD (10 μL) at 4°C under dark conditions for 5 min. Flow cytometry was performed within 30 min.

### 2.13 Autophagic Flux Assay

The mRFP‐GFP‐LC3 lentivirus vectors were used to track autophagosome formation and degradation to monitor autophagy flux. After induced by LPS and treated with MC, 16HBE cells were transfected with LC3‐mRFP‐GFP lentivirus. Single‐cell suspension was prepared from complete medium with 5 × 10^4^ cells/mL, which were seeded on adhesive slides. Autophagy was observed using laser confocal microscopy after sealed with 50% glycerol. The principle of autophagy spot detection is to label and track LC3 through mRFP (Red puncta); the strength of GFP (Green puncta) can indicate the degree of fusion between lysosomes and autophagosomes to form autolysosomes. When autophagosomes fuse with lysosomes, GFP fluorescence (Green puncta) is quenched, and only red puncta can be detected. The yellow spots appearing in the red and green puncta merge after microscopic imaging are autophagosomes, while the red puncta indicate autophagosomes. The strength of autophagic flow can be clearly observed by counting different colored puncta (Wang et al. 2021).

### 2.14 Quantitative Real‐Time PCR

TRIzol reagent was used to extract total RNA from the 16HBE cells. The RNA concentration was analyzed spectrophotometrically by absorbance readings at 260 and 280 nm. Synthesis of cDNA was done through reverse transcription (RT) using a cDNA reverse transcription kit, according to the manufacturer's recommendations and the reference (Liu et al. 2018). Primers of Kif3a, LC3B, BECN1, and caspase‐3 mRNA were designed using Primer Premier 5.0 (Table 1), and expression levels of these mRNA were detected by real‐time PCR (qPCR) using the ABI PRISM 7500 Sequence Detection System (Applied Biosystems, USA). The qPCR cycling conditions were as follows: 94°C for 2 min, followed by 30 cycles at 94°C for 30 s, 56°C for 60 s, and 72°C for 60 s. GAPDH was used as normalization, and the relative mRNA expression was calculated using the $2^{-\Delta \Delta C_{t}}$ method.

**TABLE 1:** PCR primer sequences.

Primer name	Primer sequences (5′–3′)
Kif3a forward	TCTGCCCTGGAAAAGAAGGT
Kif3a reverse	GCTTCCTCTTGCAAACTGGT
LC3B forward	GAGAGCAGCATCCAACCAAA
LC3B reverse	GACCATGCTGTGTCCGTTC
BECN1 forward	ATGCAATGGTGGCTTTCCTG
BECN1 reverse	GCTTTTGTCCACTGCTCCTC
caspase‐3 forward	ACTGGACTGTGGCATTGAGA
caspase‐3 reverse	GCACAAAGCGACTGGATGAA
GAPDH forward	TGTTGCCATCAATGACCCCTT
GAPDH reverse	CTCCACGACGTACTCAGCG

### 2.15 Western Blot Analysis

Total protein of the 16HBE cells was separated using RIPA lysis buffer. The protein concentration was detected using the BCA method. Western Blot (WB) was performed as previously described (Liu et al. 2018). The same amount, up to 30 μg of protein, was subjected to 10% SDS‐PAGE and transferred onto PVDF membranes. After being blocked with 5% skim milk, the membrane was incubated with the primary antibody for 16 h at 4°C. Later, the member was washed by PBS and incubated with horseradish peroxidase (HRP)‐conjugated secondary antibody at room temperature for 1 h. The primary antibodies were Kif3a (used at 1:800 dilution for WB), LC3B (used at 1:1000 dilution for WB), BECN1 (used at 1:1000 dilution for WB), Caspase‐3 (used at 1:1000 dilution for WB), and *β*‐actin (used at 1:1000 dilution for WB), respectively. Protein bands were detected using the chemiluminescence detection kit (ECL). The bands were semiquantified using Image J software.

### 2.16 Statistical Analysis

The data are shown as mean ± standard deviation (SD), and statistical analyses were conducted utilizing SPSS software (SPSS Inc., USA). The Least‐Significant Difference (LSD) after one‐way analysis of variance (ANOVA) was used to analyze the significant difference among groups. *p* < 0.05 was considered statistically significant.

## 3 Results

### 3.1 Effect of MC on Functional Performance in OVA‐Induced Asthmatic Rat

**FIGURE 1:**
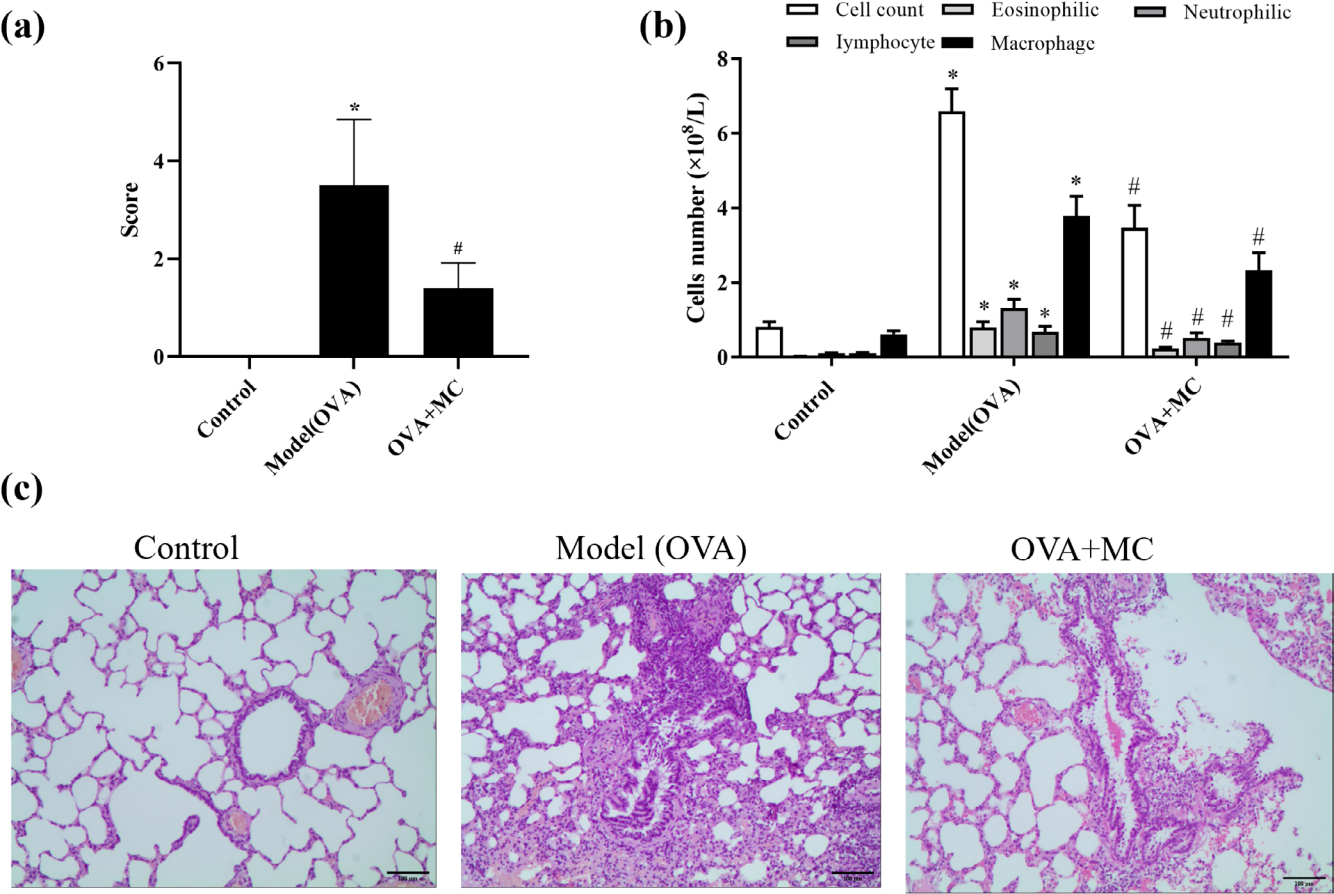
The effect of MC on functional performance in OVA‐induced asthma rats. (a) Behavioral score of rats. *p < 0.05 versus Control group; ^#^ *p* < 0.05 versus Model (OVA) group (*n* = 8). (b) The effect of MC on total cell, macrophage, neutrophil, eosinophil, and lymphocyte counts in BALF. *p < 0.05 versus Control group; ^#^ *p* < 0.05 versus Model (OVA) group (*n* = 8). (c) The effect of MC on lung tissue morphology of asthmatic rats stained with hematoxylin and eosin (HE, ×100). *p < 0.05 versus Control group; ^#^ *p* < 0.05 versus Model (OVA) group (*n* = 3).

The score of behavioral observation was shown in Table 2 and Figure 1A. The score of the model group was significantly higher than those of the normal control (*p* < 0.05); After MC treatment, its score was reduced compared with the model group (*p* < 0.05). The numbers of total cells, macrophages, neutrophils, eosinophils, and lymphocytes in BALF of rats were analyzed, as shown in Figure 1B. The numbers of total cells, macrophages, neutrophils, eosinophils, and lymphocytes were significantly elevated in the model (OVA) group compared with the control groups. Following MC treatment, these cellular indicators exhibited a marked reduction. The histological examination of lung tissue of the rat was detected, as shown in Figure 1C. In the control group, the lung tissue showed normal structure without obvious inflammation. In contrast, the model group showed exfoliation or hyperplasia of bronchial mucosal epithelial cells, more inflammatory cell infiltration in bronchus and surrounding blood vessels, obvious expansion of alveoli, and rupture of the alveolar wall. The MC group showed less bronchial epithelium exfoliation or hyperplasia, reduced inflammatory cell infiltration, and thinner alveolar walls compared to the model group. In the control group, rats in the MC group showed a reduction in epithelial shedding or hyperplasia, a decrease in inflammatory cell infiltration in lung tissue, and a reduction in alveolar wall thickness. This result indicated that MC could alleviate inflammation in OVA‐induced rats.

**TABLE 2:** Behavioral score of rats (means, *n* = 8).

Group	Normal	Model (OVA) group	OVA+MC group
1	0	3	1
2	0	3	2
3	0	3	2
4	0	3	1
5	0	3	2
6	0	6	1
7	0	3	2
8	0	6	1
Mean value	0	3.75	1.5

### 3.2 Immunohistochemistry Evaluation of MC on Kif3a, Autophagy‐Related Proteins LC3B and BECN1, and Caspase‐3 in OVA‐Induced Asthmatic Rat

**FIGURE 2:**
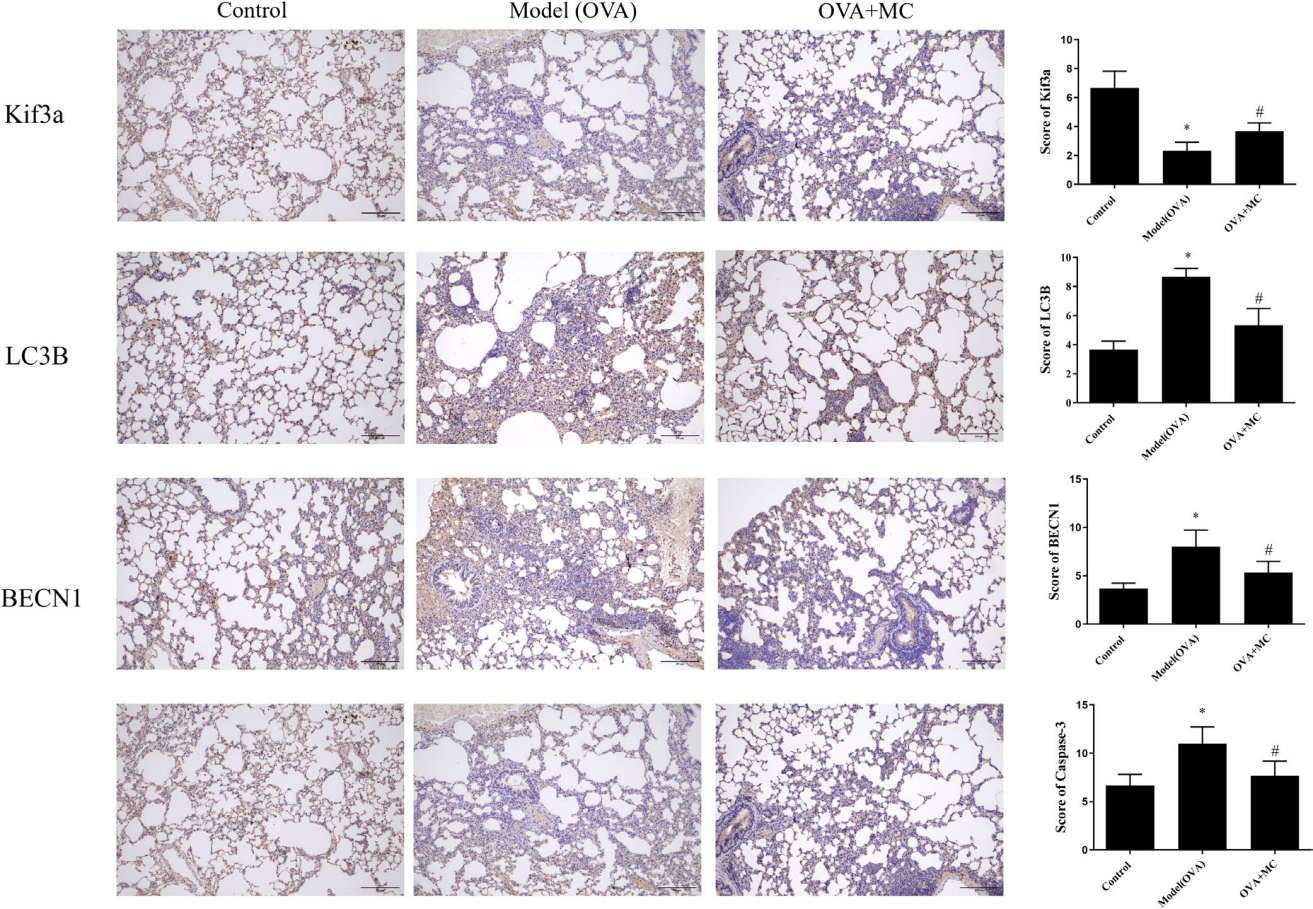
The protein expression levels of MC on Kif3a, LC3B, BECN1, and Caspase‐3 by immunohistochemistry (IHC, ×100). *p < 0.05 versus Control group; ^#^ *p* < 0.05 versus Model (OVA) group.

The protein expression levels of Kif3a, LC3B, BECN1, and Caspase‐3 were evaluated by immunohistochemistry. As shown in Figure 2, immunohistochemical analysis revealed that the protein expression of Kif3a was significantly lower in the rats from the model group compared with the control group (*p* < 0.05). However, the protein expression of LC‐3B, BECN1, and Caspase‐3 was significantly higher than that in the model group (*p* < 0.05, *p* < 0.05). After treatment of MC, the protein expression level of Kif3a was significantly increased in the MC group, and the protein expressions level of LC‐3B, BECN1, and Caspase‐3 significantly decreased the protein in the MC group, compared with the model group.

### 3.3 MC Reduces LPS‐Induced 16HBE Cell Injury

**FIGURE 3:**
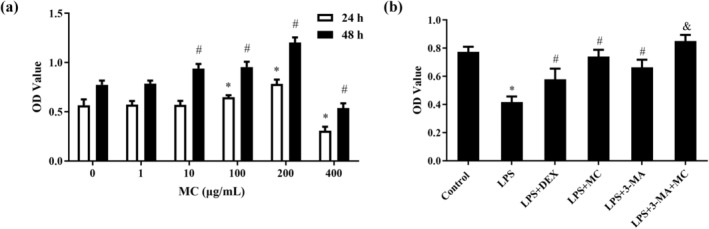
The effect of MC on cell viability. (a) The effect of MC on cell viability with different concentrations and times. (b) The effect of MC and 3‐MA on 16HBE cells induced by LPS. *p < 0.05 versus Control group; ^#^ *p* < 0.05 versus LPS group; ^&^ *p* < 0.05 versus LPS + 3‐MA group (*n* = 5).

According to previous reports, 16HBE cells were induced by 2.5 μM LPS for 16 h to establish an inflammatory cell model. Then, different concentrations of MC (0, 1, 10, 100, 200, and 400 μg/mL) were added for 24 and 48 h, respectively. 16HBE cell viability was measured with the CCK‐8 assay, and the time of treatment and concentration are shown in Figure 3A. The protective effect of MC on LPS‐induced 16HBE cell injury was dose‐dependent, increasing from 1 to 200 μg/mL and significantly decreasing at 400 μg/mL on 24 h group, compared with the model group (0 μg/mL). A similar situation was observed for the 48 h group, at the concentration range of 1–200  μg/mL, cell viability increased with the increase of MC concentrations and reached the maximum at 200 μg/mL, while the cell viability decreased significantly at the concentration of 400 μg/mL, compared with the model group (0 μg/mL). Besides, the cell viability at 48 h was higher than that at 24 h when treated with MC concentration of 200 μg/mL. Hence, the cell activity was effectively reversed by treating with MC at 200 μg/mL for 48 h, and this concentration was selected for use in the subsequent experiments. Furthermore, as shown in Figure 3B, under the same conditions, cell viability was restored when LPS‐induced 16HBE cells were pretreated with 3‐MA (3‐methyladenine, 5 mM, *p* < 0.05) for 4 h, compared with the LPS group (model group); and the LPS + 3‐MA + MC group (*p* < 0.05) had a more significant reversal effect on cell activity compared with the LPS + 3‐MA group. MC‐enhanced the protective role in the 3‐MA group, increasing cell growth viability. These results suggested that MC could effectively enhance cell viability.

### 3.4 MC Improved Apoptosis in LPS‐induced 16HBE Cells

**FIGURE 4:**
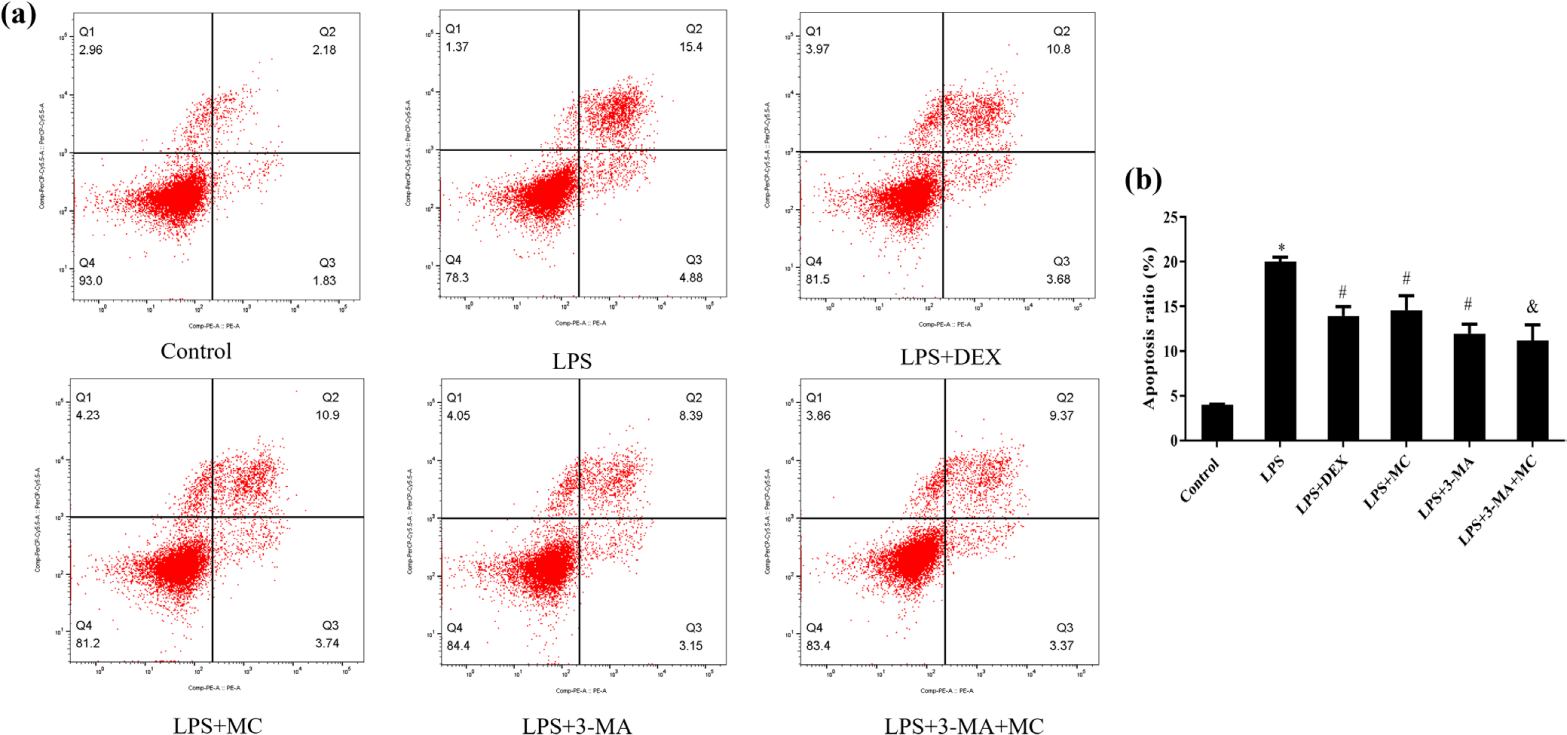
The apoptosis effect of MC against LPS‐induced 16HBE cells by flow cytometry. (a) Quadrant populations of apoptosis were measured via annexin V‐FITC/7‐AAD double staining in LPS‐induced 16HBE cells. Q1 (Annexin V+, 7‐AAD+): Dead cells; Q2 (Annexin V‐, 7‐AAD+): Late‐stage apoptosis cells; Q3 (Annexin V+, 7‐AAD‐): Early‐stage apoptosis cells; and Q4 (Annexin V‐, 7‐AAD‐): Live cells. (b) A graphical representation of the late‐stage apoptotic cells. *p < 0.05 versus the Control group; ^#^ *p* < 0.05 versus the LPS group; ^&^ *p* < 0.05 versus LPS + 3‐MA group (*n* = 3).

Apoptosis plays a role in the process of cell development, tissue homeostasis, and defense mechanisms against harmful and potentially dangerous conditions. The effect of MC on LPS‐induced apoptosis in 16HBE cells was detected using flow cytometry, as shown in Figure 4. The results showed that 2.5 μM LPS could induce apoptosis in 16HBE cells (LPS Group, *p* < 0.05), while after treatment with 200 μg/mL MC for 48 h (LPS + MC Group, *p* < 0.05), LPS‐induced apoptosis was reversed significantly. And, compared with LPS + 3‐MA, the apoptosis ratio of the group of co‐treatment with MC and 3‐MA was decreased (*p* < 0.05), significantly. This result indicated that MC could protect 16HBE cells against LPS‐induced apoptosis.

### 3.5 MC Inhibited Autophagy Flux in LPS‐induced 16HBE Cells

**FIGURE 5:**
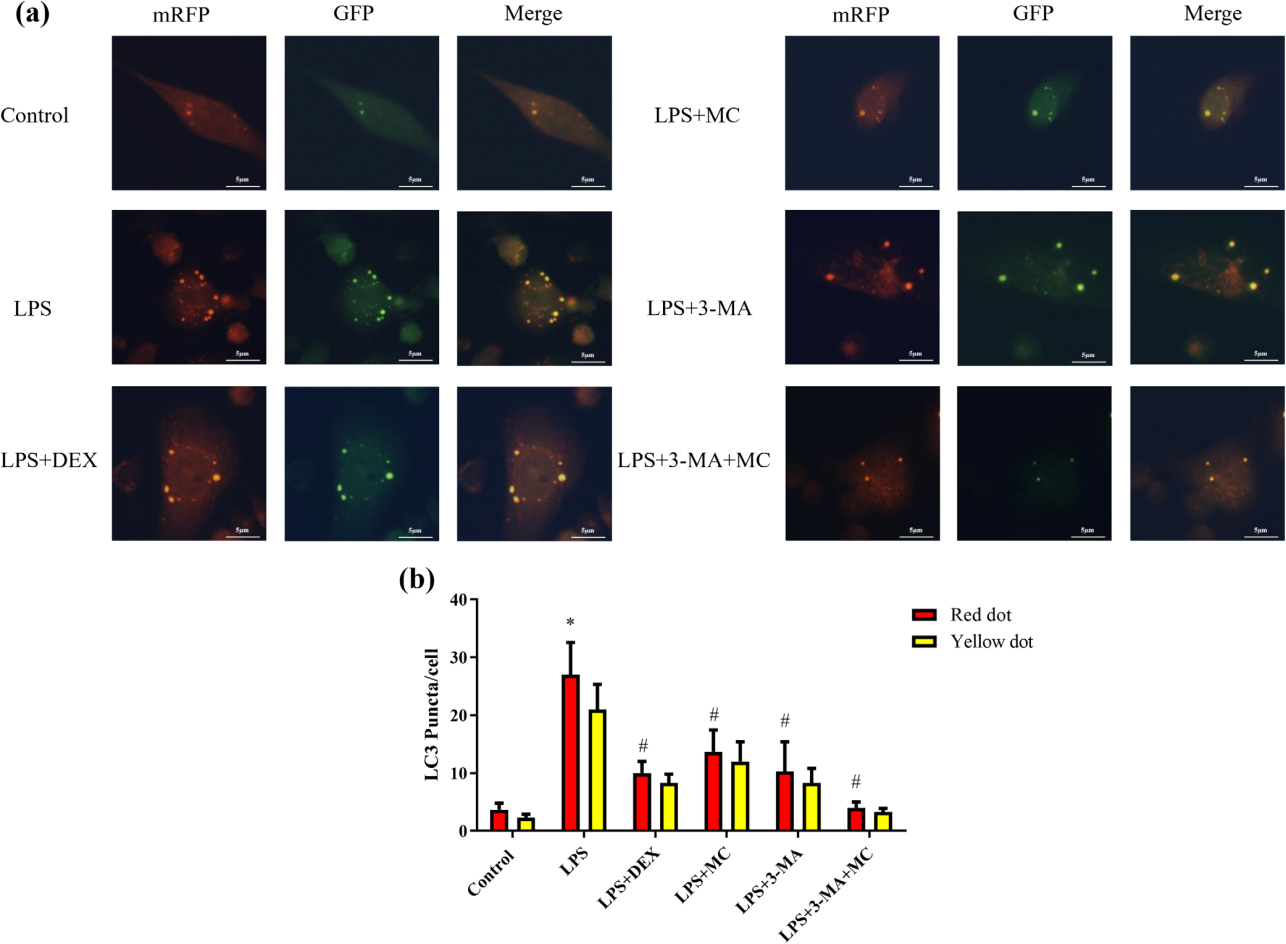
mRFP‐GFP‐LC3 distribution in LPS‐induced 16HBE cells was analyzed by confocal microscopy. (a) Fluorescence imaging of RFP and GFP in LPS‐induced 16HBE cells expressing mRFP‐GFP‐LC3. Yellow punctas point to autophagosomes and red punctas point to autolysosomes. (b) Quantification of the number of autophagosomes (yellow LC‐3 puncta) and autolysosomes (red LC‐3 puncta) per 16HBE cell.

Furthermore, 16HBE cells were transfected with an adenovirus harboring tandem fluorescent mRFP‐GFP‐LC3 to monitor the formation of autophagosomes and their matured form of autolysosomes to evaluate autophagic flux, as shown in Figure 5A. The fluorescence signals were observed under a laser confocal microscope. In neutral to basic pH, the mRFP‐GFP‐LC3 protein exhibited both mRFP fluorescence and GFP fluorescence, and the MERGE image of the GFP and mRFP, which was used to evaluate the autophagy level of MC. GFP fluorescence was rapidly quenched in a low‐pH environment, whereas mRFP fluorescence is more stable. When the autophagosomes and lysosomes eventually fused to form the autolysosomes, GFP fluorescence was quenched by lysosomal acidity and only exhibited red puncta, and the merged image appeared as red puncta; When GFP cannot be degraded by lysosomes, autolysosomes and autophagosomes all exhibited green and red fluorescence, and the merged image appeared as yellow puncta. Autophagy flux was blocked in the autophagosome‐lysosome fusion stage; a large number of yellow puncta and green puncta were observed. Hence, yellow puncta pointed to autophagosomes and red puncta pointed to autolysosomes. Quantification of the number of autophagosomes (yellow puncta) and autolysosomes (red puncta) per 16HBE cell to monitor autophagy flux. As shown in Figure 5B, normal 16HBE cells exhibited basal autophagy; however, the red puncta and yellow puncta of 16HBE cells induced by LPS increased significantly in the LPS model group (*p* < 0.05), indicating that autophagy and autophagic flux were enhanced. After treatment with MC for 48 h, the red puncta and yellow puncta were significantly decreased compared with the model (LPS) group. 3‐MA, known as a typical autophagy inhibitor, suppressed autophagic activity. Our result consisted with LPS + DEX group and LPS + 3‐MA group. With the intervention of 3‐MA and MC (LPS + 3‐MA + MC group, *p* < 0.05), the red puncta and yellow puncta were significantly reduced in 16HBE cells compared with LPS + 3‐MA group. These results indicated that MC has a synergistic effect with 3‐MA, which can inhibit autophagy and restore autophagy flow to normal levels.

### 3.6 Effect of MC on Expression Levels of Kif3a, LC3B, BECN1, and Caspase‐3 mRNA in LPS‐Induced 16HBE Cells

**FIGURE 6:**
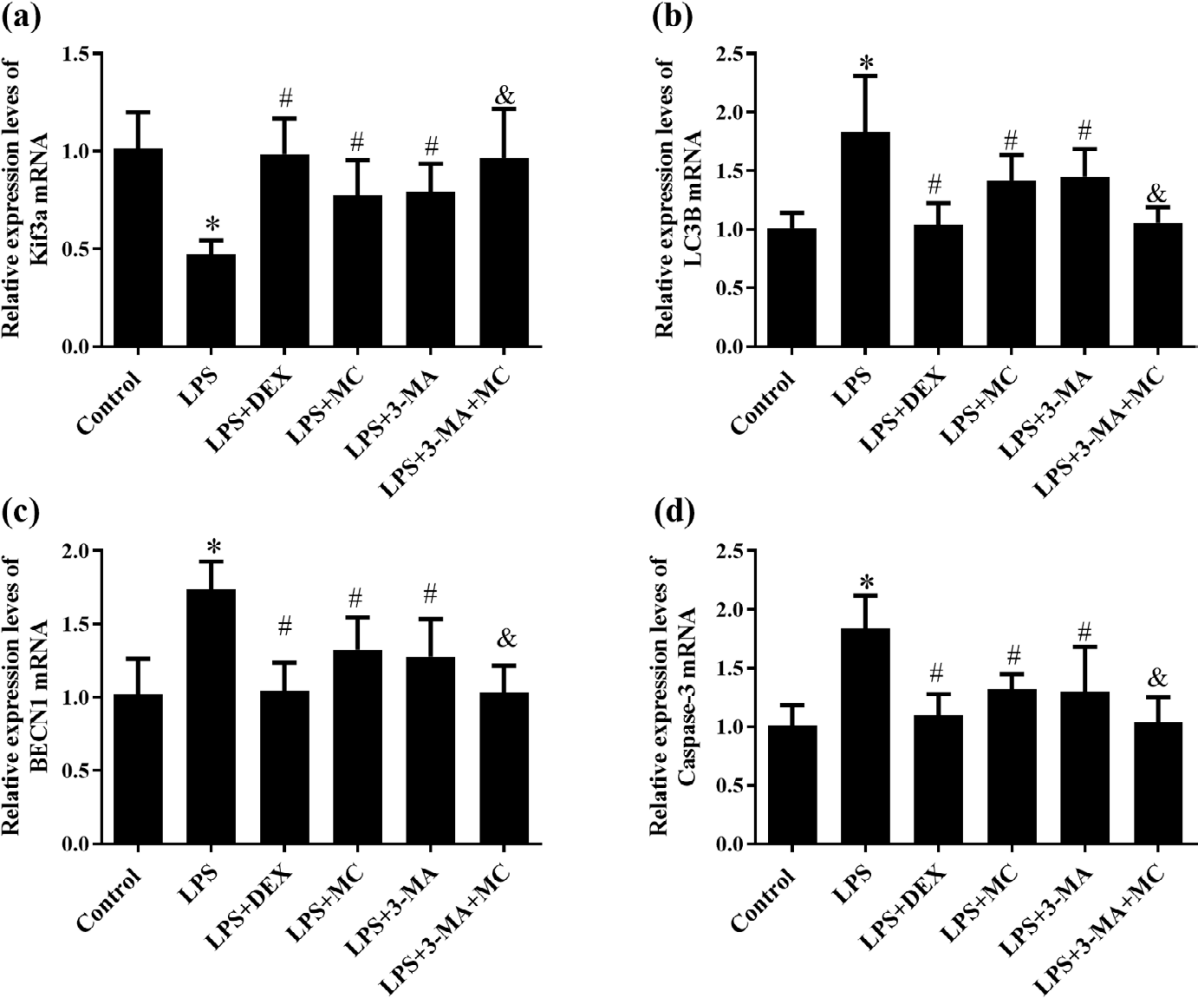
The relative gene expression levels of MC in LPS‐induced 16HBE cells on (a) Kif3a, (b) LC3B, (c) BECN1, and (d) Caspase‐3. *p < 0.05 versus Control group; ^#^ *p* < 0.05 versus LPS group; ^&^ *p* < 0.05 versus LPS + 3‐MA group.

The kinesin family number 3A (Kif3a) is a subunit of the kinesin‐2 motor protein that regulates microtubule function and trafficking, which is one of the susceptibility sites of asthma (Geng et al. 2018). High expression of LC3B and BECN1 in 16HBE cells can induce autophagy and even autophagic cell death, Caspase‐3 is also one of the key proteins involved in apoptosis. Therefore, we performed reverse‐transcription‐polymerase chain reaction (PCR; RT‐PCR) detection on the above four indicators to further evaluate the anti‐asthma effect of MC, as shown in Figure 6. After 16HBE cells were induced by LPS, the mRNA expression of Kif3a was decreased (*p* < 0.05), the mRNA expression of Caspase‐3 was increased (*p* < 0.05), and the mRNA expression of autophagy‐related LC3B and BECN1 was increased in the model group (*p* < 0.05, *p* < 0.05). The MC could reverse the above changes, promote the mRNA expression of Kif3a, and decrease the mRNA expression of Caspase‐3, LC3B, and BECN1; these benefits were increased significantly by a pretreatment with the autophagy inhibitor 3‐MA, and MC remarkably synergized with 3‐MA to reduce the autophagy level (LPS + 3‐MA + MC group, *p* < 0.05). Hence, LPS stimulated 16HBE cells to be at the level of over‐activated autophagy, which resulted in apoptosis death, and MC can down‐regulate gene expression on apoptosis and autophagy and promote Kif3a expression to protect 16HBE cells damaged by LPS.

### 3.7 Effect of MC on Expression Levels of Kif3a, Autophagy‐Related Proteins LC3B and BECN1, and Caspase‐3 in LPS‐Induced 16HBE Cells

**FIGURE 7:**
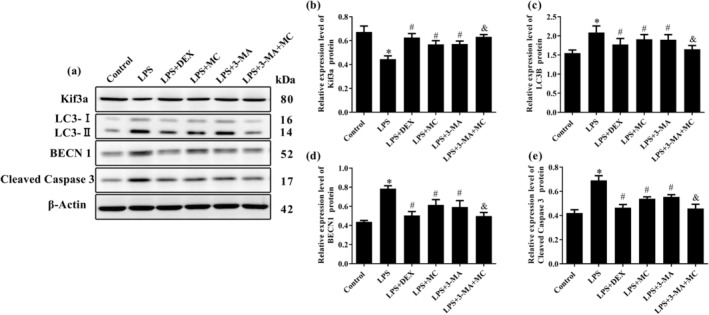
The effect of relative protein levels of MC in LPS‐induced 16HBE cells on (a) protein bands and protein expression levels of (b) Kif3a, (c) LC3B, (d) BECN1, and (e) Cleaved Caspase‐3. *p < 0.05 versus the Control group; ^#^ *p* < 0.05 versus the LPS group; ^&^ *p* < 0.05 versus the LPS + 3‐MA group (*n* = 3).

To investigate the effect of MC in LPS‐stimulated 16HBE cells, the western blot assay was used to analyze the protein levels of Kif3a, LC3B, BECN1, and Cleaved Caspase‐3. As shown in Figure 7, Compared with control group, the protein levels of Kif3a were decreased (*p* < 0.05) and the protein levels of LC3B‐II/I, BECN, and Cleaved Caspase‐3 (*p* < 0.05, *p* < 0.05, *p* < 0.05) were increased in the LPS group. After treatment with MC, the protein levels of Kif3a, LC3B‐II/I, BECN1, and Caspase‐3 (*p* < 0.05, *p* < 0.05, *p* < 0.05, *p* < 0.05) were improved compared with LPS group, which was consistent with the changes of intracellular proteins in LPS‐induced 16HBE cells after 3‐MA pretreatment. In addition, with treatment of 3‐MA and MC, these protein expression levels were regulated significantly (LPS + 3‐MA + MC group, *p* < 0.05), compared with LPS + 3‐MA group. Thus, we concluded that MC down‐regulates LPS‐induced apoptosis and autophagy‐related protein expression and increases Kif3a expression to attenuate LPS‐induced airway inflammation in 16HBE cells.

## 4 Discussion

Asthma is a chronic inflammatory disease of the respiratory tract, which causes airway inflammation due to damage of airway epithelium, and then appears repeated wheezing, shortness of breath, cough, and other symptoms (Boonpiyathad et al. 2019). Active ingredients from natural edible plants have advantages in relieving asthma symptoms, controlling asthma attacks, and preventing asthma recurrence, which have become the focus of researchers (Zhou et al. 2017). *M. chamomile* is one kind of medical and edible plant with an anti‐inflammatory effect. It is one of the most popular flower tea ingredients on the market for the prevention of inflammation and has attracted the attention of researchers in recent years (Li et al. 2023). Chamomile was discovered as a novel and selective COX‐2 inhibitor that possesses anti‐inflammatory activity (Srivastava, Pandey, and Gupta 2009), and its oil had anti‐hyperglycemic and anti‐inflammatory effects through targeting inflammatory cytokines and the NF‐κB/NLRP3 signaling pathway (Saghahazrati et al. 2020). Chamomile extract can accelerate the healing of traumatic oral ulcers by reducing epithelial cell apoptosis and TNF‐α expression in diabetic rats (Oliveira et al. 2016). Besides, we previously reported the flavonoids were the important bioactive components from MC, mainly including luteolin, luercetin, kaempferol, and their related compounds, which act against asthma through reducing antioxidant and anti‐inflammatory effects to improve asthma symptoms (Li et al. 2023; Qiao et al. 2023); And, we have found that MC could reduce the IgE level and increase glutathione peroxidase (GSH‐Px) in the serum of OVA‐induced rats to ameliorate OVA‐induced lung injury (Li et al. 2023). Thus, in this study, we mainly focused on exploring the mechanism of MC on anti‐asthma.

The OVA‐induced asthma model is a representative asthma animal model that resembles human asthma (Min et al. 2020). An OVA‐evoked asthma model using SD rats is useful to detect the efficacy of MC on allergic asthma (Thakur et al. 2019). As a result of OVA sensitization and boosting, asthmatic rats showed classical shortness of breath, panting, wheezing, and sneezing, and, total and differential cell counts of leukocytes in BALF were significantly increased compared with the control groups. In addition, exfoliation or hyperplasia of bronchial mucosal epithelial cells, more inflammatory cell infiltration in the bronchus and surrounding blood vessels, obvious expansion of alveoli, and rupture of the alveolar wall were observed in the OVA group. These results were similar to previous findings (Gu et al. 2016; Min et al. 2020). Following MC treatment, improvements were observed in behavioral parameters, total and differential leukocyte counts in bronchoalveolar lavage fluid (BALF), inflammatory cell infiltration, as well as lung and bronchial tissue and structural integrity. These findings are consistent with those reported in previous studies (Li et al. 2023). We observed the possible beneficial antiasthma effects of MC on the OVA‐induced asthma rat.

Previously, we found that *M. chamomile* had an anti‐asthma effect through the target protein Kif3a using proteomic technology (Chang et al. 2020). Kif3a is crucial for lung function, aiding in cilia formation and maintenance for mucociliary clearance and influencing lung cell morphology, homeostasis, and repair via autophagy and apoptosis pathways. Kif3a expression was lower in nasal and bronchial epithelia in the asthmatic model, and overexpressing Kif3a reduced epithelial cell apoptosis and bronchial inflammation (Geng et al. 2018). In vitro, knocking down Kif3a increased epithelial apoptosis, boosted CCL17, IL‐5, and IL‐8 transcription, and raised COX‐2 protein levels and β‐catenin translocation; Conversely, Kif3a overexpression greatly alleviates asthma (Geng et al. 2018). Our results were consistent with previous reports that Kif3a was significantly decreased in the model (OVA) group compared with the control group. After treatment of MC, the Kif3a level was significantly increased compared with the model group.

Autophagy is a self‐degrading process that helps maintain cellular homeostasis, which is closely correlated with the severity of asthma (Liu et al. 2016). A recent study found that asthma patients have higher autophagy levels compared to healthy individuals (Ban et al. 2016). Autophagy‐related genes, such as ATG5, Beclin‐1 (BECN1), and LC3, are involved in and regulate the pathogenesis of asthma (Huang et al. 2022). Our study was consistent with the previous report that the level of autophagy was hyperactivated in asthmatic rats after OVA stimulation (Liu et al. 2016). The level of autophagy of LC3B and BECN1 could be restored by MC intervention. Besides, it has been previously reported that excessive autophagic activation could trigger autophagic programmed cell death (Pi et al. 2019). We also had a similar finding that Caspase‐3 levels were significantly increased in the lung tissue of OVA‐stimulated asthmatic rats and significantly improved after MC treatment. Hence, MC could play an anti‐asthma role by up‐regulating Kif3a level, inhibiting autophagy, and apoptosis.

Airway epithelium is the first line of defense in many respiratory diseases, particularly in asthma. Bronchial epithelial cells play an important role in maintaining airway homeostasis of the respiratory system, while proinflammatory mediators were released from damaged 16HBE cells to aggravate asthma inflammation (Song et al. 2023). Lipopolysaccharide (LPS), the main component of gram‐negative bacteria, is a biologically active ingredient in cigarette smoke. Airway inflammation caused by adverse factors such as LPS can lead to damage and dysfunction of airway epithelial cells, thereby exacerbating the pathogenesis of respiratory diseases including asthma (Sun et al. 2019). Hence, the LPS‐induced 16HBE cell model was the common cell model for asthma airway inflammation, and the effect of MC on viability was tested in LPS‐induced 16HBE cell inflammation model. The viability of 16HBE cells was decreased significantly after induced with LPS, while MC could enhance the viability of 16HBE cells in the dose‐dependent manner. Besides, the LPS‐induced 16HBE cells viability was increased significantly at the concentration of MC of 200 μg/mL for 48 h. MC played a protective role for LPS‐induced 16HBE cells.

LC3 and Becline‐1 (BECN 1) are important factors for autophagy (Liu et al. 2016). BECN1 serves a critical function in the nucleation process of autophagy. LC3 exists in the form of LC3‐I and LC3‐II, and LC3‐II is involved in the formation of double‐membrane vesicles and the extension of the autophagosome membrane and is considered a biomarker of autophagy initiation (Dong et al. 2023). 3‐MA is a classical inhibitor of autophagy, which mainly inhibits the formation and development of autophagosomes (Silveira et al. 2020). In this study, we combined with 3‐MA to investigate the protective effect of MC on asthma through autophagy. Dexamethasone (DEX) is a pharmacological agent that has a well‐known clinical effect on the improvement in lung function in asthma; therefore, we used DEX as a positive control. Cytotoxicity experiments showed that MC and 3‐MA enhanced viability of 16HBE cells injured by LPS, which was consistent with the DEX positive group. The results demonstrate that LPS treatment promoted apoptosis in the16HBE cells, which was validated by flow cytometry (Xia, Yang, and Zhu 2023), and MC and 3‐MA could reduce cell apoptosis, respectively. Interestingly, cell viability and apoptosis were significantly improved when 16HBE cells were treated with 3‐MA and MC, compared with the 3‐MA group. MC could enhance the protective effect of 3‐MA, indicating that MC could be involved in the autophagy process to protect against LPS damage of 16HBE cells. Furthermore, LC3 puncta were observed by confocal microscopy to evaluate autophagosome formation (Ying et al. 2017). As previously reported, autophagy was overactivated in 16HBE cells by LPS stimulation, while MC and 3‐MA could inhibit excessive autophagy, and the inhibition effect was more significant when co‐treated with 3‐MA and MC. These results indicated that the overactivated autophagy proteins of BECN1 and LC3‐II induced by LPS were decreased via treatment with MC, both at the gene and protein levels. Besides, it has been previously reported that excessive autophagic activation could trigger autophagic programmed cell death (Pi et al. 2019). MC could reverse LPS‐induced apoptosis by reducing Cleaved Caspase‐3 levels at both gene and protein levels. The efficacy of 3‐MA and MC in attenuating the LPS‐induced asthma was similar. MC could inhibit hyperactivation of autophagy and apoptosis in 16HBE cells induced by LPS.

Kif3a is not only a susceptibility gene locus associated with asthma but also an important kinesin required for ciliogenesis that is known to transduce key extracellular signals, including the sonic hedgehog (SHH) pathway (Giridhar et al. 2016). Kif3a is required for the formation of primary cilia, which are controlled by autophagy (Tang et al. 2013). Autophagy can transport Kif3a to lysosomes for degradation through autophagosomes, and 3‐MA blocked the autophagosome formation and sustained adequate resources of proteins for primary cilia generation (Wang et al. 2015). Besides, Kif3a has also been involved in apoptosis. Studies showed that overexpression of Kif3a could efficiently suppress the increased epithelia apoptosis of nasal mucosa and bronchia and bronchial inflammation (Geng et al. 2018). Studies have found that the formation of autophagosomes can promote the activation of caspase and induce cell apoptosis (Young et al. 2012). We found that the expression of Kif3a was decreased after stimulation with LPS in 16HBE cells, while this was significantly reversed by MC, supporting by qRT‐PCR and western blot analyses. After co‐treatment of 3‐MA and MC, the expression of Kif3a was higher than that of LPS + 3‐MA group, and the apoptosis ratio, autophagy flow, and the expression of BECN1, LC3‐II, and Caspase‐3 were lower than that of LPS + 3‐MA group, and tended to be normal. Hence, MC is a potential autophagy inhibitor, which could up‐regulate the level of Kif3a and down‐regulate the level of Caspase‐3 through inhibiting the level of excessive activation of autophagy to exert an anti‐asthma effect. The findings suggest that *M. Chamomile* exhibits substantial potential as a novel therapeutic agent or as an ingredient in medicinal foods for the treatment of asthma. Nevertheless, the relationship between Kif3a and autophagy is intricate, as Kif3a is essential for primary cilia formation and plays a regulatory role in autophagy (Tang et al. 2013). In the next step, we will study the regulatory effect of *M. Chamomile* on autophagy by knocking down or overexpressing KIF3A levels in cellular and animal asthma models. The effect of *M. Chamomile* on improving asthma inflammation through KIF3A and autophagy will be further clarified, providing a theoretical basis for the clinical development and application of *M. Chamomile* in the treatment of asthma.

## 5 Conclusion

In this study, the OVA‐induced asthma rat model and LPS‐induced 16HBE cells model were used to evaluate the effect of the active fraction of *M. Chamomile* against asthma. *M. Chamomile*, as a potential autophagy inhibitor, could inhibit over‐activated autophagy levels, increase Kif3a expression, and decrease apoptosis to ameliorate asthma. This study explores the protective mechanism of *M. Chamomile* in asthma treatment and provides a basis for its development as the pharmaceutical agent and/or dietary supplement for asthma management.

## Author Contributions

**Jun Peng:** funding acquisition (supporting), investigation (equal), methodology (equal), software (equal), validation (equal), writing – original draft (equal). **Feicui Zhao:** formal analysis (equal), funding acquisition (supporting), investigation (equal), methodology (equal), visualization (equal). **Xiaolong Kang:** formal analysis (equal), investigation (equal), validation (equal). **Nadire Aierken:** data curation (equal), validation (equal). **Qian Li:** conceptualization (equal), funding acquisition (lead), investigation (equal), methodology (equal), project administration (equal), resources (equal), supervision (equal), writing – review and editing (equal).

## Ethics Statement

This study was approved by the Institutional Animal Care and Use Committee of Xinjiang Medical University (approval number: IACUC‐20240227‐75).

## Conflicts of Interest

The authors declare no conflicts of interest.

## Data Availability

The data that support the findings of this study are available on request from the corresponding author. The data are not publicly available due to privacy restrictions.

## References

[fsn370030-bib-0001] Al‐Dabbagh, B. , I. A. Elhaty , M. Elhaw , et al. 2019. “Antioxidant and Anticancer Activities of Chamomile (*Matricaria recutita* L.).” BioMed Research Notes 12, no. 1: 3. 10.1186/s13104-018-3960-y. PMC6317209 30602390

[fsn370030-bib-0002] Ban, G. Y. , D. L. Pham , T. H. Trinh , et al. 2016. “Autophagy Mechanisms in Sputum and Peripheral Blood Cells of Patients With Severe Asthma: A New Therapeutic Target.” Clinical and Experimental Allergy 46, no. 1: 48–59. 10.1111/cea.12585. 26112695

[fsn370030-bib-0003] Barnes, P. J. , J. Baker , and L. E. Donnelly . 2022. “Autophagy in Asthma and Chronic Obstructive Pulmonary Disease.” Clinical Science 136, no. 10: 733–746. 10.1042/cs20210900. 35608088 PMC9131388

[fsn370030-bib-0004] Boonpiyathad, T. , Z. C. Sozener , P. Satitsuksanoa , and C. A. Akdis . 2019. “Immunologic Mechanisms in Asthma.” Seminars in Immunology 46: 101333. 10.1016/j.smim.2019.101333. 31703832

[fsn370030-bib-0005] Carrozza, D. , G. Malavasi , E. Ferrari , and M. C. Menziani . 2023. “Alginate Beads Containing Cerium‐Doped Mesoporous Glass and Curcumin: Delivery and Stabilization of Therapeutics.” International Journal of Molecular Sciences 24, no. 1: 880. 10.3390/ijms24010880. 36614324 PMC9821038

[fsn370030-bib-0006] Chakaya, J. , and N. Aït‐Khaled . 2022. “Global Asthma Report 2022: A Wake‐Up Call to Enhance Care and Treatment for Asthma Globally.” International Journal of Tuberculosis and Lung Disease 26, no. 11: 999–1000. 10.5588/ijtld.22.0483. 36281047

[fsn370030-bib-0007] Chang, Y. D. , C. H. Li , C. H. Tsai , Y. W. Cheng , J. J. Kang , and C. C. Lee . 2020. “Aryl Hydrocarbon Receptor Deficiency Enhanced Airway Inflammation and Remodeling in a Murine Chronic Asthma Model.” Federation of American Societies for Experimental Biology Journal 34, no. 11: 15300–15313. 10.1096/fj.202001529R. 32959404

[fsn370030-bib-0008] Chou, M. C. , I. M. Jou , H. T. Chen , and R. Chang . 2023. “Traditional Chinese Medicine Use May Reduce Medical Utility in Patients With Asthma: Correspondence.” Quarterly Journal of Medicine 116, no. 3: 256. 10.1093/qjmed/hcac088. 35471660

[fsn370030-bib-0009] Cvetanovic, A. , J. Svarc‐Gajic , Z. Zekovic , et al. 2015. “Comparative Analysis of Antioxidant, Antimicrobiological and Cytotoxic Activities of Native and Fermented Chamomile Ligulate Flower Extracts.” Planta 242, no. 3: 721–732. 10.1007/s00425-015-2308-2. 25976264

[fsn370030-bib-0010] Dong, H. , W. Yang , W. Li , et al. 2023. “New Insights Into Autophagy in Inflammatory Subtypes of Asthma.” Frontiers in Immunology 14: 1156086. 10.3389/fimmu.2023.1156086. 37090692 PMC10117973

[fsn370030-bib-0011] El Mihyaoui, A. , J. C. G. Esteves da Silva , S. Charfi , M. E. Candela Castillo , A. Lamarti , and M. B. Arnao . 2022. “Chamomile (*Matricaria chamomilla* L.): A Review of Ethnomedicinal Use, Phytochemistry and Pharmacological Uses.” Life (Bethesda) 12, no. 4: 479. 10.3390/life12040479. PMC9032859 35454969

[fsn370030-bib-0012] Fei, L. , Rahima·Abdulla , X. Xuelei , and L. Qian . 2024. “Study on Extraction Technology of Anti‐Asthma Active Part of *Matricarla chamomilla* L.” China Journal of Traditional Chinese Medicine and Pharmacy 39, no. 3: 1528–1534.

[fsn370030-bib-0013] Geng, G. , Y. Du , J. Dai , D. Tian , Y. Xia , and Z. Fu . 2018. “KIF3A Knockdown Sensitizes Bronchial Epithelia to Apoptosis and Aggravates Airway Inflammation in Asthma.” Biomedicine & Pharmacotherapy 97: 1349–1355. 10.1016/j.biopha.2017.10.160. 29156524

[fsn370030-bib-0014] Giridhar, P. V. , S. M. Bell , A. Sridharan , et al. 2016. “Airway Epithelial KIF3A Regulates Th2 Responses to Aeroallergens.” Journal of Immunology 197, no. 11: 4228–4239. 10.4049/jimmunol.1600926. PMC5123825 27794000

[fsn370030-bib-0015] Gu, W. , R. Cui , T. Ding , et al. 2016. “Simvastatin Alleviates Airway Inflammation and Remodelling Through Up‐Regulation of Autophagy in Mouse Models of Asthma.” Respirology 22, no. 3: 533–541. 10.1111/resp.12926. 27782356

[fsn370030-bib-0016] Huang, C. , M. Peng , J. Tong , et al. 2022. “Vitamin D Ameliorates Asthma‐Induced Lung Injury by Regulating HIF‐1alpha/Notch1 Signaling During Autophagy.” Food Science & Nutrition 10, no. 8: 2773–2785. 10.1002/fsn3.2880. 35959262 PMC9361460

[fsn370030-bib-0017] Janarny, G. , K. Gunathilake , and K. Ranaweera . 2021. “Nutraceutical Potential of Dietary Phytochemicals in Edible Flowers‐A Review.” Journal of Food Biochemistry 45, no. 4: e13642. 10.1111/jfbc.13642. 33533514

[fsn370030-bib-0018] Jiao, B. , Y. Chen , Y. Yang , et al. 2021. “Toluene Diisocyanate‐Induced Inflammation and Airway Remodeling Involves Autophagy in Human Bronchial Epithelial Cells.” Toxicology In Vitro 70: 105040. 10.1016/j.tiv.2020.105040. 33127434

[fsn370030-bib-0019] Li, F. X. , and J. Liu . 2020. “Research Progress in Drug Treatment of Refractory Bronchial Asthma.” Medical Review 26, no. 8: 1573–1577.

[fsn370030-bib-0020] Li, Q. , R. Abdulla , X. L. Xin , et al. 2023. “Profiling of Chemical Constituents of *Matricarla chamomilla* L. by UHPLC‐Q‐Orbitrap‐HRMS and In Vivo Evaluation Its Anti‐Asthmatic Activity.” Heliyon 9, no. 5: e15470. 10.1016/j.heliyon.2023.e15470. 37153405 PMC10160356

[fsn370030-bib-0021] Li, Q. , J. Lu , and J. Li . 2017. “Effect of n‐Butanol Extract of *M. chamomile* on Asthma Model Mice.” Chinese Traditional Patent Medicine 39, no. 12: 2603–2606.

[fsn370030-bib-0022] Li, Q. , X. Xin , and A. Haji Akber . 2021. “Study on Extraction Technology of Total Flavonoids From *Matricarla chamomilla* L.” China Journal of Traditional Chinese Medicine and Pharmacy 36, no. 4: 2342–2344.

[fsn370030-bib-0023] Liu, J. N. , D. H. Suh , H. K. T. Trinh , Y. J. Chwae , H. S. Park , and Y. S. Shin . 2016. “The Role of Autophagy in Allergic Inflammation: A New Target for Severe Asthma.” Experimental & Molecular Medicine 48, no. 7: e243. 10.1038/emm.2016.38. 27364893 PMC4973311

[fsn370030-bib-0024] Liu, L. , M. Yasen , D. Tang , J. Ye , H. A. Aisa , and X. Xin . 2018. “Polyphenol‐Enriched Extract of *Rosa rugosa* Thunb Regulates Lipid Metabolism in Diabetic Rats by Activation of AMPK Pathway.” Biomedicine & Pharmacotherapy 100: 29–35. 10.1016/j.biopha.2018.01.143. 29421579

[fsn370030-bib-0025] Long, Y. , H. Wang , Z. Ma , et al. 2023. “Combined Epimedii Folium and Ligustri Lucidi Fructus With Dexamethasone Alleviate the Proliferation of Airway Smooth Muscle Cells by Regulating Apoptosis/Autophagy.” Journal of Ethnopharmacology 314: 116547. 10.1016/j.jep.2023.116547. 37178983

[fsn370030-bib-0026] Lv, X. , K. Li , and Z. Hu . 2020. “Asthma and Autophagy.” Advances in Experimental Medicine and Biology 1207: 581–584. 10.1007/978-981-15-4272-5_41. 32671776 PMC7360921

[fsn370030-bib-0027] Min, B. G. , S. M. Park , Y. W. Choi , et al. 2020. “Effects of Pelargonium Sidoides and Coptis Rhizoma 2 : 1 Mixed Formula (PS + CR) on Ovalbumin‐Induced Asthma in Mice.” Evidence‐Based Complementary and Alternative Medicine 2020, no. 1: 9135637. 10.1155/2020/9135637. 32190091 PMC7066403

[fsn370030-bib-0028] Mota, S. T. S. , L. Vecchi , D. A. Alves , et al. 2020. “Annexin A1 Promotes the Nuclear Localization of the Epidermal Growth Factor Receptor in Castration‐Resistant Prostate Cancer.” International Journal of Biochemistry & Cell Biology 127: 105838. 10.1016/j.biocel.2020.105838. 32858191

[fsn370030-bib-0029] Neves, J. M. , C. Matos , C. Moutinho , G. Queiroz , and L. R. Gomes . 2009. “Ethnopharmacological Notes About Ancient Uses of Medicinal Plants in Tras‐Os‐Montes (Northern of Portugal).” Journal of Ethnopharmacology 124, no. 2: 270–283. 10.1016/j.jep.2009.04.041. 19409473

[fsn370030-bib-0030] Oliveira, B. V. , P. G. de Barros Silva , J. D. Nojosa , et al. 2016. “TNF‐Alpha Expression, Evaluation of Collagen, and TUNEL of *Matricaria recutita* L. Extract and Triamcinolone on Oral Ulcer in Diabetic Rats.” Journal of Applied Oral Science 24, no. 3: 278–290. 10.1590/1678-775720150481. 27383710 PMC5022216

[fsn370030-bib-0031] Pi, H. , M. Li , L. Zou , et al. 2019. “AKT Inhibition‐Mediated Dephosphorylation of TFE3 Promotes Overactive Autophagy Independent of MTORC1 in Cadmium‐Exposed Bone Mesenchymal Stem Cells.” Autophagy 15, no. 4: 565–582. 10.1080/15548627.2018.1531198. 30324847 PMC6526814

[fsn370030-bib-0032] Qiao, X.‐r. , T. Feng , D. Zhang , et al. 2023. “Luteolin Alleviated Neutrophilic Asthma by Inhibiting IL‐36γ Secretion‐Mediated MAPK Pathways.” Pharmaceutical Biology 61, no. 1: 165–176. 10.1080/13880209.2022.2160770. 36604842 PMC9828607

[fsn370030-bib-0033] Saghahazrati, S. , S. A. Ayatollahi , F. Kobarfard , and B. Minaii Zang . 2020. “Attenuation of Inflammation in Streptozotocin‐Induced Diabetic Rabbits by *Matricaria chamomilla* Oil: A Focus on Targeting NF‐kappaB and NLRP3 Signaling Pathways.” Chinese Herbal Medicines 12, no. 1: 73–78. 10.1016/j.chmed.2019.12.003. 36117563 PMC9476470

[fsn370030-bib-0034] Silveira, J. S. , G. L. Antunes , D. B. Kaiber , et al. 2020. “Autophagy Induces Eosinophil Extracellular Traps Formation and Allergic Airway Inflammation in a Murine Asthma Model.” Journal of Cellular Physiology 235, no. 1: 267–280. 10.1002/jcp.28966. 31206674

[fsn370030-bib-0035] Singh, O. , Z. Khanam , N. Misra , and M. K. Srivastava . 2011. “Chamomile (*Matricaria chamomilla* L.): An Overview.” Pharmacognosy Reviews 5, no. 9: 82–95. 10.4103/0973-7847.79103. 22096322 PMC3210003

[fsn370030-bib-0036] Song, Y. , W. Fu , Y. Zhang , et al. 2023. “Azithromycin Ameliorated Cigarette Smoke‐Induced Airway Epithelial Barrier Dysfunction by Activating Nrf2/GCL/GSH Signaling Pathway.” Respiratory Research 24, no. 1: 69. 10.1186/s12931-023-02375-9. 36879222 PMC9990325

[fsn370030-bib-0037] Srivastava, J. K. , M. Pandey , and S. Gupta . 2009. “Chamomile, a Novel and Selective COX‐2 Inhibitor With Anti‐Inflammatory Activity.” Life Sciences 85, no. 19: 663–669. 10.1016/j.lfs.2009.09.007. 19788894 PMC2784024

[fsn370030-bib-0038] Sun, J. , N. Huang , W. Ma , H. Zhou , and K. Lai . 2019. “Protective Effects of Metformin on Lipopolysaccharide‐Induced Airway Epithelial Cell Injury via NF‐kappaB Signaling Inhibition.” Molecular Medicine Reports 19, no. 3: 1817–1823. 10.3892/mmr.2019.9807. 30628691

[fsn370030-bib-0039] Tang, Z. , M. G. Lin , T. R. Stowe , et al. 2013. “Autophagy Promotes Primary Ciliogenesis by Removing OFD1 From Centriolar Satellites.” Nature 502, no. 7470: 254–257. 10.1038/nature12606. 24089205 PMC4075283

[fsn370030-bib-0040] Thakur, V. R. , V. Khuman , J. V. Beladiya , K. K. Chaudagar , and A. A. Mehta . 2019. “An Experimental Model of Asthma in Rats Using Ovalbumin and Lipopolysaccharide Allergens.” Heliyon 5, no. 11: e02864. 10.1016/j.heliyon.2019.e02864. 31768443 PMC6872797

[fsn370030-bib-0041] Wang, S. , M. J. Livingston , Y. Su , and Z. Dong . 2015. “Reciprocal Regulation of Cilia and Autophagy via the MTOR and Proteasome Pathways.” Autophagy 11, no. 4: 607–616. 10.1080/15548627.2015.1023983. 25906314 PMC4502771

[fsn370030-bib-0042] Wang, Z. , Q. Wu , C. Li , S. Sun , Z. Li , and J. Wu . 2021. “Chapter 12—Quantitative Determination of Autophagy Flux by Probes.” In Methods in Cell Biology, edited by O. Kepp and L. Galluzzi , vol. 164, 157–165. Academic Press. 10.1016/bs.mcb.2021.02.002 34225913

[fsn370030-bib-0043] Xia, F. , L. Yang , and X. F. Zhu . 2023. “Knockdown of circ_0038467 Alleviates Lipopolysaccharides‐Induced 16HBE Cell Injury by Regulating the miR‐545‐3p/TRAF1 Axis in Neonatal Pneumonia.” Microbial Pathogenesis 179: 106091. 10.1016/j.micpath.2023.106091. 37045695

[fsn370030-bib-0044] Xu, Q. , W. Liu , X. Liu , et al. 2016. “Silibinin Negatively Contributes to Primary Cilia Length via Autophagy Regulated by Histone Deacetylase 6 in Confluent Mouse Embryo Fibroblast 3T3‐L1 Cells.” Molecular and Cellular Biochemistry 420, no. 1–2: 53–63. 10.1007/s11010-016-2766-2. 27435857

[fsn370030-bib-0045] Xu, Z. H. , Y. Y. Gao , H. T. Zhang , K. F. Ruan , and Y. Feng . 2018. “Progress in Experimental and Clinical Research of the Diabetic Retinopathy Treatment Using Traditional Chinese Medicine.” American Journal of Chinese Medicine 46, no. 7: 1–27. 10.1142/S0192415X1850074X. 30284463

[fsn370030-bib-0046] Yin, Q. , S. Hou , H. Yin , et al. 2019. “The Analgesic and Anti‐Inflammatory Effects of Zukamu Granules, a Traditional Chinese Medical Formulation.” Pharmaceutical Biology 57, no. 1: 729–735. 10.1080/13880209.2019.1675716. 31794281 PMC6913673

[fsn370030-bib-0047] Ying, L. , G. J. Zhao , Y. Wu , et al. 2017. “Mitofusin 2 Promotes Apoptosis of CD4(+) T Cells by Inhibiting Autophagy in Sepsis.” Mediators of Inflammation 2017: 4926205. 10.1155/2017/4926205. 29358849 PMC5735308

[fsn370030-bib-0048] Young, M. M. , Y. Takahashi , O. Khan , et al. 2012. “Autophagosomal Membrane Serves as Platform for Intracellular Death‐Inducing Signaling Complex (iDISC)‐Mediated Caspase‐8 Activation and Apoptosis.” Journal of Bone and Joint Surgery 287, no. 15: 12455–12468. 10.1074/jbc.M111.309104. PMC3320995 22362782

[fsn370030-bib-0049] Zhou, F.‐F. , Z.‐X. Xu , A. Adila , and J.‐Y. Li . 2017. “Recent Research Progress in Immunomodulatory Effects of Chinese Herbal Medicine on Asthma Treatment.” China Journal of Chinese Materia Medica 42, no. 19: 3713–3717. 10.19540/j.cnki.cjcmm.20170907.006. 29235284

